# Nutritional Composition and Environmental Impact of Meals Selected in Workplace Canteens before and after an Intervention Promoting the Adherence to the Mediterranean Diet

**DOI:** 10.3390/nu14214456

**Published:** 2022-10-23

**Authors:** Alice Rosi, Beatrice Biasini, Elisa Monica, Valeria Rapetti, Valeria Deon, Francesca Scazzina

**Affiliations:** 1 Human Nutrition Unit, Department of Food and Drugs, University of Parma, 43125 Parma, Italy; 2 Global Nutrition & Wellbeing Unit, Research, Development & Quality Group, Barilla G. e R. Fratelli, 43122 Parma, Italy

**Keywords:** sustainable menus, Mediterranean Diet, environmental burden, educational intervention, choice architecture, worksite cafeteria, blue collars, office workers, caterer

## Abstract

Enhancing healthy and sustainable food systems is one of the key goals of the current European Commission policy. In this light, the creation of a food environment where people are properly informed about the healthiness and sustainability of food choices is essential. This study aimed to evaluate the nutritional profile and the environmental impact of meals consumed in a workplace canteen in Italy in the presence of a nudge (i.e., the Double Pyramid logo) combined with a web-based application promoting the Mediterranean Diet. Energy and nutrient contents and the carbon, water, and ecological footprints of 29,776 meals were compared across three subsequent periods (from June to April) through one-way ANOVA. Although the choice of dishes labelled with the Double Pyramid logo was comparable across periods, the selection of fish- and plant-based dishes increased from +2% (fish, vegetables) up to +17% (whole-grain cereals), with a concurrent reduction of meat-based options (−2%). Although the consumption of healthy items increased (*p* < 0.001), they were not added as a replacement for alternative options, leading to a higher content in energy (*p* < 0.001) and nutrients (*p* < 0.001) and worse environmental footprints, contrarily to what was observed when data were adjusted for energy. The intervention significantly improved food choices; however, as the higher selection of desired dishes was not adequately compensated for, it was not fully effective.

## 1. Introduction

Providing workers with nutritious, safe, and affordable food is considered a public health strategy [1]. As reported by the Italian National Institute for Insurance against Accidents at Work (INAIL), a proper diet for workers must take into account both energy and nutrient contents of the meals consumed during the day, the type of work performed (i.e., sedentary, varied, light, heavy) and the number of working hours, the physical conditions of the environment in which the work is carried out (e.g., temperature, humidity, etc.), the food habits (i.e., cultural background), and extra work activities (e.g., sports, second job, hobbies) [2]. To avoid a decrease in attention span and other cognitive functions, it is also important to minimise the risk of hypoglycaemia that can occur when meals are skipped [3]. On the other hand, meals eaten at work should not exceed the proper energy and nutrient amount, mainly in the case of sedentary work, during which energy expenditure is limited. Moreover, they should be easily digestible, mainly consisting of whole grains, legumes, fruit and vegetables, and should provide adequate hydration [2,4]. 

Such food composition reflects the planetary health plate suggested by the EAT-Lancet Commission, which identifies a flexitarian dietary model as globally healthy and environmentally sustainable, taking into account a multitude of scientific targets affected by food production activities [5]. Plant-based meals, with eventual small quantities of meat, fish, and dairy products, are healthy and eco-friendly options, and their habitual consumption within a balanced energy diet contributes to reducing the mortality and disease risk as well as mitigating negative externalities caused by food systems, such as resource exploitation, terrestrial acidification, water pollution, deforestation, climate change and biodiversity loss [5,6]. Therefore, following a sustainable diet is an objective and an essential means to achieve a sustainable food system [7]. 

To quantify food system and diet pressure on the environment, several indicators have been used. Among these, the carbon footprint (which accounts for greenhouse gas (GHG) emissions) = and the use of water, land or energy have been extensively investigated in relation to the production of single food products [6], their consumption within meals [8], and actual and alternative dietary patterns, including the Mediterranean Diet (MD) [9,10]. 

The MD is an example of a flexitarian diet, referring to the traditional dietary pattern adopted in the southern Italy population in the 1960s [11]; it has widely emerged as a dietary model linked to both health and environmental benefits. Specifically, the evidence of positive health outcomes has been found to be convincing in reducing the risk for cardiovascular diseases, diabetes and metabolic syndrome, highly suggestive in relation to breast cancer and cognitive function, and suggestive for total mortality, overweight and obesity, and certain types of cancers [12]. From the environmental perspective, the MD has been associated with reduced resource use and lower GHG emissions compared to current population diets [9]. Furthermore, the inextricable interaction fostered by the new MD pyramid between food and diet, conviviality, gastronomic culture, agriculture, traditions, and other lifestyle dimensions (such as physical activity and rest) has contributed to the evolution of the MD concept from a healthy to a sustainable eating pattern [13]. 

Despite the increasing awareness from the political and scientific perspective about the importance of the food environment in determining food intake, little work has been conducted on analyzing the availability of healthy food in specific scenarios, especially in worksite settings [14]. 

Given that moving towards more healthy and sustainable food systems is one of the key goals of the European Green Deal, promoting a food environment where people are properly informed about the healthiness and sustainability of their food choices is paramount [15]. People are more inclined to change their eating behaviors if they are gently guided in their decision-making process. Although healthy eating nudges have been extensively used [16], a few studies have analyzed the effectiveness of nudges in increasing healthy and sustainable food chosen and consumed out of the home [17,18]. As defined by Thaler and Sunstein [19], a nudge is “any aspect of the choice architecture that alters people’s behavior in a predictable way without forbidding any options or significantly changing their economic incentives”. Strictly speaking, when applied in food services, nudges refer to the menu redesign, the alteration of placement or portion size of dishes, or the use of signs, symbols, labels, or claims aimed at moving food choice.

To foster more conscious food choices, the Barilla Centre for Food and Nutrition (BCFN) developed the “Double Pyramid”, an intuitive educational tool that combines the food pyramid, inspired by the Mediterranean Diet, and the environmental pyramid models [20]. The model demonstrates a very close relationship between the nutritional value of a food and the environmental impact generated during its production and consumption, measured as the ecological footprint. This graphic tool suggests that, in principle, foods that should be consumed more frequently (i.e., plant-based foods) present a lower ecological footprint compared to the products whose intake should be moderate (i.e., animal-based food). 

Therefore, the objective of the present study was to evaluate the nutritional profile and the environmental impact of meals served and consumed in a worksite canteen setting by the company employees in the presence of the “Double Pyramid” nudge combined with a web-based application designed to promote the adherence to the Mediterranean Diet. Specifically, the nutritional analysis of the menus aims to evaluate energy and nutrient contents, whereas carbon, water, and ecological footprints are assessed to determine the environmental impacts of menus. 

The project follows a previous nutritional intervention programme enhancing the MD, which was implemented in the same setting a few years earlier but without the inclusion of the web-based tool associated with a rewards program [21]. The former intervention was able to effectively improve the dietary choices of employees in the canteens, but during the intervention phase, both nutritionists and the canteen staff were actively involved, making the intervention not fully bearable over time, contrary to the web-based application that could be used for a prolonged period.

## 2. Materials and Methods

### 2.1. Study Design

A longitudinal study design was applied. The study took place in the two worksite canteens of the Barilla G & R. F.lli S.p.A. food company, based in Pedrignano (Parma, Italy), from June 2018 to April 2019. All canteen users (both genders, 19–67 years old, blue-collar or office workers) were invited to take part in the study by means of information sent to employees through the administration’s profiling database and by exhibiting specific flyers in the worksite canteens.

Since 2011, the *sì.mediterraneo* healthy eating education project has been carried out at the company headquarters in Pedrignano (Parma, Italy), where more than 1500 employees work. The primary aims of the *sì.mediterraneo* project are (i) increasing the awareness of employees about the benefits of the Mediterranean Diet and the relationship between proper nutrition and environmental sustainability through nutritional information and education in line with the principles of the Double Pyramid promoted by the BCFN, and (ii) improving the eating habits through a wider, more varied, healthy and sustainable offer of Mediterranean dishes available in the company’s canteens. 

Access to the canteens at the time of the study was open to all the employees from 12.00 to 14.30 for lunch. One of the two cafeterias was also open for dinner from 18.30 to 20.00. The company employees were offered a complete lunch menu, free of charge, composed of three dishes such that the employee can choose between a selection of first courses (i.e., dishes composed mainly of starchy foods, such as pasta, rice, etc.) and/or second courses (i.e., dishes mainly composed of protein-based foods, such as meat, fish, eggs, dairy products, legumes) or main dishes (i.e., dishes composed of both starchy and protein-based foods, such as mixed salads), side dishes (i.e., vegetables) and fruit. Additional servings and beverages or desserts were instead paid for by the employees. However, no price promotion for specific recipes was applied, as all dishes in each category (i.e., first course, second course, main dish, side dish, dessert, beverage) were offered at the same (relatively low) price. The menus are based on a 4-week rotation and are differentiated across the four seasons to guarantee the offer of seasonal fresh fruit and vegetables. Most of the meals offered at the worksite canteens were freshly prepared every day using mostly fresh ingredients (e.g., seasonal fruits and vegetables). A few dishes were instead prepared using canned or frozen ingredients (e.g., fish, pulses, vegetable burgers).

A nudge technique was applied beginning in 2011 and throughout the whole study duration, as the healthy and sustainable dishes within the list of the canteen menus were marked every day with the Double Pyramid logo showing the recommended ones. In 2011, to improve the availabilities of some foods, new legume-based dishes were added to menus, and some existing recipes were adjusted to improve both the nutritional profile and the environmental impact. Furthermore, the weekly frequency of specific dishes (e.g., French fries, ham, etc.) was also reduced to offer healthier choices. During the observation period (from June 2018 to April 2019), the menus did not undergo any additional changes. The logo was applied to the first courses, second courses and main dishes, which were compliant with specific energy, nutritional and environmental sustainability criteria. The nutritional criteria were defined as follows: energy ≤ 500 kcal/serving, fibre ≥ 6 g/serving, and saturated fatty acids < 3 g/serving for first courses; energy ≤ 600 kcal/serving and saturated fats ≤ 2.5 g/serving for main courses and saturated fats ≤ 2.5 g/serving for second courses. As an environmental criterion, the ecological footprint threshold applied to all courses was < 5 global m^2^/serving.

In addition, the canteen environment was set up with posters reporting information about: (1) the Mediterranean Diet; (2) the recommended consumption frequencies of food groups; (3) the food’s environmental impact and the indicators used to measure it.

The new *sì.mediterraneo* project started in September 2018 with the aim to improve the dietary habits of employees using new educational materials also delivered through a specifically designed web application. The canteen users had the option to freely register to the web-based application promoting healthy food choices based on the Mediterranean dietary model. The digital tool provided employees with educational resources on the Mediterranean Diet and the nutritional and environmental impacts of dietary choices. The tool was integrated with employees’ cafeteria cards, tracking their food choices at the worksite canteens, converting them into nutritional and carbon footprint values and providing feedback on participants’ choices. In addition, the tool was integrated with a chatbot that suggested the best menu of the day considering the offer of the canteen and the choices made by the user in the previous days to promote a varied diet and the frequency of consumption of food groups based on the Mediterranean dietary pattern. The web app also had a rewards scheme based on the actual choices made in the canteen that gave points if adherent to the nutritional and environmental recommendations.

### 2.2. Data Collection

All meals bought and consumed at the canteen site for lunch and/or dinner were recorded at baseline (time 0, from June to September 2018), time 1 (from September to December 2018), and time 2 (from January to April 2019). Specifically, the analysis considered a total of 50 working days (from Monday to Friday) for each 3-month time period and excluded days around holidays (e.g., Christmas and Easter) due to the non-representativeness of the menus served at the canteen and the limited number of users. Only subjects registered to the web-based program who purchased at least three menus in at least one period were included in the data collection. All subjects were exposed to the Double Pyramid nudge starting from t0. During the intervention period, some employees left, others joined the company, and some were temporally absent during certain days due to working travel and personal or holiday leaves. 

Data were collected automatically by using the personal employee’s badge connected to the cash register, where cashiers typed the purchased products. Through the electronic system, the food selected by the canteen users was recorded in terms of quality (which food items) and quantity (number of portions) consumed for each digital transaction.

Only menus representative of the participants’ meal consumption pattern and providing at least 300 kcal were considered. This minimum energy threshold was set based on the definition according to which a meal is represented by any food intake occasion providing at least 15% of the total energy (with 2000 kcal/day taken as reference for the average adult energy requirement), regardless of the quality of consumed food [22]. Multiple consecutive transactions associated with one user on the same day were merged if it was clear they corresponded to a unique meal (e.g., if one transaction included only fruit or beverages, it was merged with the previous one on the same day). 

### 2.3. Nutritional and Environmental Evaluation

Actual food consumption was estimated, assuming that the selected meal was entirely consumed by the subject. On this basis, the amount (i.e., grams/millilitres) of food items consumed for each meal was estimated from each digital transaction based on the dish recipes, without any ex post recipe modifications due to the discretionary use of seasonings to the dishes (i.e., extra-virgin olive oil, vinegar, salt, etc.). 

The *Tool Chef* software, a digital tool of the company used only for internal analysis, linked to the *sì.mediterraneo* one, was used to calculate the number of portions of each food group and the number of items with the Double Pyramid logo selected per menu, as well as the nutritional composition and the environmental footprints of the meals. In particular, each recipe was linked to a specific number of portions of food groups based on its ingredients. The standard portions considered were: 1 plate of a first course (corresponding to an average of 80 g of raw pasta or cereals), 50 g of bread and 30 g of bread substitutes, 1 plate of a second course (e.g., 150 g of fish, 100 g of meat, 50 g of cured meat, 100 g of fresh cheese, 50 g of dried legumes), 200 g of vegetables (80 g for salad), 150 g of fruit, 330 mL of beverages (e.g., sugared or sugar-free beverages), and 125 mL of fruit juices [23]. 

The *Tool Chef* software contains data from the Food Composition Database for Epidemiological Studies in Italy [24], used to evaluate the mean energy and nutrient composition of the meals. In particular, the content of energy, proteins, fats, saturated fatty acids, total carbohydrates, sugars, fibre, and salt was calculated for each consumed meal by summing the values of each ingredient used to prepare the recipes of each food item. 

Similarly, the *Tool Chef* software includes the BCFN’s environmental impact database [20] used to assess the environmental impact of each meal consumed. Three indicators were retrieved from the environmental database: carbon (g of CO_2_ equivalent emissions), water (L) and ecological (m^2^ of biologically productive land and water needed to regenerate the consumed resources and to absorb the emissions/waste) footprints. Environmental values took into account the cooking methods (e.g., boiling, baking) applied during meal preparation. For each meal, the three environmental footprints were estimated by summing the relative performance of single ingredients of each dish.

### 2.4. Statistical Analysis 

The statistical analyses were performed by the SPSS software (IBM Corp. Released 2020. IBM SPSS Statistics for Macintosh, Version 27.0. Armonk, NY: IBM Corp), setting the significance at *p* < 0.05. Data are presented as mean ± standard deviation or as numbers and percentages. Percentages of dishes labelled with the Double Pyramid logo and of food groups consumed in the canteens were obtained by dividing the number of dishes/portions by the number of total meals recorded for each period, multiplied by 100. 

The chi-squared test was used to explore associations among time periods for dish percentages, whereas differences in the number of dishes labelled with the Double Pyramid logo and of portions consumed for each food group were investigated by applying a non-parametric Kruskal–Wallis test with pairwise comparisons. Similarly, a parametric one-way ANOVA with the Tamhane post hoc test was applied to assess differences in the energy, nutritional and environmental composition of meals. 

## 3. Results

A total of 401 employees took part in the study (47% females, 44.2 ± 10.9 years). 

The choice of dishes labelled with the Double Pyramid logo was similar among the three time periods (*p* = 0.107), with a median of 1 (25–75th percentile: 1–2) labelled dish per meal in all periods, and with 99% of meals having at least one labelled dish during the whole study duration (data not shown).

**Figure 1:**
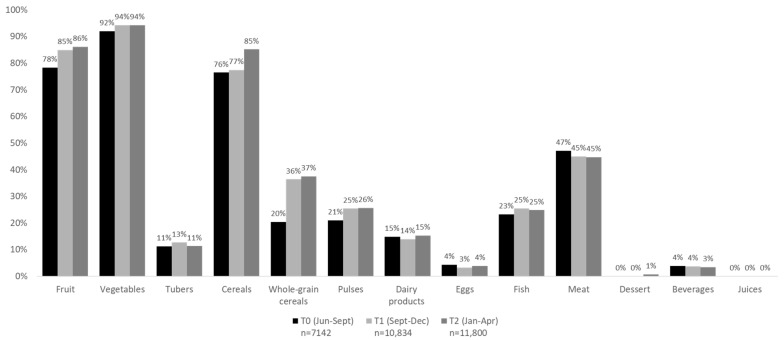
Frequencies of choice of each food group by time periods (T0: baseline from June to September; T1: time period 1 from September to December; T2: time period 2 from January to April).

Changes in the frequencies of selection of each food group among time periods are shown in Figure 1. The frequencies of choice of a portion of fruit (78%), vegetables (92%) and cereals (76%) were high at baseline. However, an increased selection of +7% at Time 1 and +8% at Time 2 for fruit, +2% for vegetable portions at both periods, and +1% at Time 1 and +9% at Time 2 for cereals was registered. The numbers of portions of whole-grain cereals were the most increased, with +16% and +17% at Time 1 and Time 2, respectively, compared to baseline. Additionally, portions of pulses increased at Time 1 (+4%) and Time 2 (+5%), fish portions reached +2%, while meat decrease accounted for −2% at both time periods.

Similarly, considering the composition of meals in terms of the portion of food groups (Supplementary Table S1), an increase in portion per tray of fruit, whole grain cereals, and pulses but also desserts was observed from baseline to Time 1 and Time 2 (*p* < 0.001). The portions per tray of refined cereals and dairy products were higher at Time 2 with respect to baseline and Time 1 (*p* < 0.001), whereas the selection of fish, eggs and tubers was greater at Time 1 than baseline and Time 2 (*p* < 0.001). A decrease in portions per meal of meat was observed from baseline to Time 1 and Time 2 (*p* = 0.020). The number of portions per tray of vegetables, beverages, and juice was similar among time periods. 

In line with the previous results, when the number of selected portions was transformed into categorical variables (Table 1), significant associations were found between the amount of food groups consumed per meal and the three time periods, except for beverages and juice, the intake of which was extremely low across the time periods. Interestingly, the share of trays containing at least one portion of fruit increased from 68% (baseline) to 75% (at both Time 1 and Time 2), while the proportion of meals containing at least one portion of whole grain cereals increased from 18% (baseline) to 31% at Time 1 and 32% at Time 2. The percentage of trays containing at least one portion of pulses switched from 17% (baseline) to 20% and 21% at Time 1 and Time 2, respectively, with a concurrent decrease (from 79% to 72% and 74%) in the share of legume-free transactions. Similarly, a small increase in the percentage of meals with at least a portion of fish (from 20% at baseline to 23% at Time 1 and 21% at Time 2) and a portion of vegetables (66% at baseline and 75% at both Time 1 and Time 2) was observed, whereas around 38% of trays with at least a portion of meat were selected in each time period.

The increased number of portions per tray observed for some food groups was also reflected in a generally higher intake of energy and nutrients during the intervention phases compared to baseline (Table 2). The highest energy, protein, fat, carbohydrate, fiber, and salt intakes were registered at Time 2, while the lowest was observed at baseline. However, energy contribution from proteins was higher at baseline than at Time 1 and Time 2; the percentage of energy from fat remained similar among time periods, whereas energy from carbohydrates and fiber increased during the intervention phases compared to baseline. The saturated fat intake was lower at baseline and similar between Time 1 and Time 2, but energy contribution from this nutrient was similar among the three time periods. The highest sugar intake and the highest energy contribution from sugars were observed at Time 1, while the lowest ones were observed Sat baseline. 

The increased selection of some food groups during the intervention phases also influenced the environmental impacts of selected meals (Table 3). Carbon and water footprints increased over time, with the highest impacts registered at Time 2 and the lowest ones registered at baseline. The lowest ecological footprint was also observed at baseline, but values were similar between Time 1 and Time 2. In contrast to these results, when environmental data were expressed per 1000 kcal, the highest footprints were observed for all three environmental indicators at baseline, while they were similar between intervention phases (Time 1 and Time 2).

## 4. Discussion

This longitudinal study described the effect of an educational intervention based on a nudge technique coupled with a web-based program aimed at promoting healthy and environmentally sustainable food choices in two worksite canteens. The intervention was offered at no cost to all employees of the Barilla G & R. F.lli S.p.A. food company who had access to the canteens. 

Due to the very high proportion of trays containing at least one dish marked with the Double Pyramid logo at the baseline, the nudge tool showed no added effect in improving the food choices when combined with additional educational materials. This could also be due to the high quality of served meals that are focused on the promotion of healthy and sustainable food choices. A similar nudge showing only the MD pyramid was applied in a previous study by Vitale and colleagues [21] to highlight the healthy plates resembling the traditional MD. However, similarly to the present study, the effectiveness of the MD Pyramid logo was not tested as a separate intervention compared to the baseline condition. Thus, the evaluation of a direct association between the food choice improvement and the sole logo application was not possible. Different choice architectures suggested for the food services can be found in the literature, such as modifying portion sizes, increasing the prominence of healthy options, and including and positioning symbols/labels to highlight healthy and/or sustainable options (e.g., vegetarian offers). When the approaches showed some ability to shift decisions, consumers were often found to be unaware about the influence exerted on their choices [25]. However, specifically referring to the use of symbols/labels, no univocal results in terms of efficacy have been observed. Stressing the fact that a dish is “vegetarian” or “free from meat” has proven to be less effective compared to the use of other framing options [26]. Specifically, the use of the “V” symbol placed before or after the titles of vegetarian dishes did not exert a significant influence on food choice in an online study involving UK consumers [27]. On the other hand, descriptive name labels referring to sustainability have proven to effectively influence decisions in a university canteen setting [17], suggesting a higher level of attractiveness for such descriptions compared to the healthy/nutritional dimension. 

Positive results related to the choice of plant-based products were observed across the study, as the selection of fruit, pulses, and whole-grain cereals, followed by vegetables, increased over time, underlining the effectiveness of the *sì.mediterraneo* educational intervention. On the other hand, more heterogeneous results can be observed for animal-based food groups. Indeed, although the choice of fish seemed to slightly increase and a small reduction in the selection of meals containing meat was observed, the share of meat-based dishes constantly remained almost double, three times and more than ten times the share of meals containing, respectively, one portion of plant-protein sources (i.e., pulses), dairies, and eggs.

Our results appear to be consistent with what was observed in an online randomised controlled trial [27], suggesting that, to allow the large-scale selection of vegetarian dishes, the offer of these dishes should widely overcome that of meat options (75% vs. 25%). However, mixed findings have emerged from the literature, as a prior study [28] proved that a perfect balance between meat and vegetarian (50% vs. 50%) options was enough to entail a relevant shift in vegetarian options in four dish menus. In this light, it would be important to increase the offer of plant-based protein alternatives in the canteens since it was lower than the animal-based options. 

From the nutritional perspective, the average energy content of the meals was comprised between 32% at baseline and 35–36% (during the intervention periods) of the adult energy requirement used as reference (2000 kcal/die). The energy intake observed at baseline was slightly lower compared to the share of energy the lunch should provide daily (i.e., 35–40%) [4]. Therefore, the increase in energy intake observed during the intervention phases should be considered a positive change in participants’ diet composition. The carbohydrate content of lunch meals was adequate as it provided 51–52% of energy intake. Indeed, according to the Italian dietary guidelines [23], the daily share of energy due to carbohydrates should equal 45–60%. The energy contribution to the meals of fat, which was around 26–27%, was consistent with the proportion of energy (20–35%) that should derive from fat daily [23]. Nevertheless, the actual fat intake, mainly derived from extra virgin olive oil, could be higher due to the possible dressings made directly by users at the table, especially for side dishes. Last, the proportion of energy from proteins, which corresponded to 21–22%, appears to be slightly high.

The significant increase in the energy content of the selected meals seems to be particularly driven by sugars, proving that, on average, a higher fruit selection was not compensated for by a reduction in the choice of other food groups. Even if the higher selection of desired dishes did not adequately replace the choice of other options, the average sugar content of the chosen meals remained below the recommended 15% of the meal energy content [23] during the three time periods. In addition, the fiber content was satisfying, accounting for 40–48% of the recommended daily fiber intake (i.e., 25 g) for the Italian adult population [23]. Promisingly, even better achievements were reported by Lassen and colleagues [29] in a study carried out in 15 Danish worksite canteens. Indeed, after a 10-year period, in parallel to an increase in the estimated mean intake of fruit and vegetables from lunch meals, the authors observed a reduction in the energy density, with no concurrent reduction in the portion size of the meals. 

An important finding of the study relates to the remarkably and increasingly high level of salt in the dishes offered at the canteens across the time periods. As shown, on average, the amount of salt in each tray was approximately equal to one-third of the daily average salt consumption in the Italian population (about 9 g) [30] and more than two-thirds of the maximum daily recommended amount (5 g) [4]. It is worth noting that the actual salt intake derived from the canteen dishes could be even higher due to the discretionary salt possibly added by canteen users at the table. This observation confirms previous considerations on the need to simultaneously develop consumer-based and establishment-based strategies [31] addressed to the different figures acting in the food services, ranging from the canteen staff to the final consumers, to maximise beneficial outcomes. 

As practical implications of the present study, tailored training sessions dedicated to cooks can be suggested to improve the nutritional quality of dishes, as already reported in the same setting by Vitale and colleagues (2017). Furthermore, it is worth noting that, to effectively improve the dietary intake in workplace canteens, even if further evidence is needed, a multitude of actions should be applied, ranging from the application of consumer-based strategies to the modification of the meal offering to enhancing the proposal of healthier options while limiting the counterparts [31].

From the environmental perspective, for all three considered indicators, the intervention did not lead to a reduction in the environmental burden associated with the chosen meals. This finding is consistent with the increased food consumption across time. However, when data were adjusted by energy, the carbon, water, and ecological footprints were lower compared to the baseline levels, suggesting that the higher absolute values could have been due to a higher amount of food selected across the three time periods, but the food choices were slightly shifted towards more sustainable dietary patterns. On average, the selected meals were characterised by a relatively high carbon emission intensity and water consumption, accounting, respectively, for more than 1000 g of CO_2_ eq and more than 1000 L of water per meal. Indeed, such ideal cut-offs have been suggested in the framework of the SU-Eatable project led by the BCFN [32] as a scenario in line with a 1.5 °C–2 °C increase by 2100 [5], applying impact values of food items selected from Petersson and colleagues (2021) [33]. 

The lunch meals’ GHG emissions seem to be consistent with the calculation made for the Italian diet, as estimated from the national consumption survey (INRAN-SCAI 2005–2006), accounting for 3.6 kg CO_2_ eq, calculated as the average impact of females’ (3.2 kg CO_2_ eq) and males’ (4.0 kg CO_2_ eq) food intake [34]. Similarly, in a more recent study involving an omnivorous Italian sample, the estimated carbon footprint was 3.9 kg CO_2_ eq [35]. Thus, GHG emissions of served meals in the present study covered around 28–30% of the average daily Italian values. Consistently, meals contributed to around 35% of both water and ecological footprints compared to the Italian population values reported in the literature [34,35].

Furthermore, if compared to the results obtained from an LCA study carried out on about 20 million meals served in 240 Swiss canteens in 2011, the mean carbon footprint calculated per lunch meal is about one-fourth (1.1 CO_2_ eq in our study vs. 4.1 kg CO_2_ eq) [36]. Such discrepancies may be explained, however, from a different data specificity characterizing the two studies. Our environmental impact analysis took into account the food production phase and the contribution estimated for different cooking typologies and duration. The Swiss study [36] instead considered all stages from farm to the transport to the canteen, including the estimated contribution derived from packaging, transportation, refrigeration, waste disposal, as well as the resource use linked to the activities in the canteen.

The environmental database used, even if regularly updated and based on information from the scientific literature, cannot be considered complete, representing one of the limitations of the present analysis. In addition, the lack of a baseline scenario where the nudge was not present can be considered a study limitation, as it prevented the possibility of investigating the single effect of the applied nudge compared to a control condition. Furthermore, the food selection was considered a proxy of actual food consumption, which was not more directly estimated as no waste records were collected. Despite the prospective study design, the same subject was not specifically followed over time, hindering the assessment of intra-subject variability during the 10 months of observation. Moreover, as only the sex and age of participants were recorded during data collection, in-depth assessments of choice determinants cannot be carried out. Additional socio-demographic information, such as education and income levels, could be relevant as a heterogeneous population is considered in this study, with both white and blue collars being involved. However, the experimental conditions, such as the relatively low price at which the meals are offered at the company canteens, contributed to minimising the influence of economic factors.

Last, it should be taken into consideration that these results may not be totally generalizable to the general working population, as all participants are employees of a food company where the double logo pyramid has been created, and they may have more knowledge on these topics than the general population. The adoption of a certain food behaviour (e.g., healthy and sustainable food choice) results from a combination of intrinsic (e.g., food preferences, personal attitude and/or motivation and/or knowledge) and extrinsic factors (i.e., the food environment, food policy framework) that singularly can variably affect food intake [7,37]. Therefore, the inclusion of additional outcome variables would be beneficial to investigate the so-called “attitude-behaviour gap”, which reflects the discrepancy between a favourable attitude toward a certain behaviour and the behaviour itself [38].

Despite these considerations, to the best of our knowledge, this is the first explorative analysis of the dietary and environmental contents of meals served in worksite canteens in Italy. The main strengths of the study relate to the high number of transactions that were qualitatively and quantitatively assessed during the study, which allowed us to capture a reliable overview of the food choice made at the worksite canteens over an extended period (10 months).

## 5. Conclusions

This study shows the positive effect of an education program applying a nudge intervention represented by the Double Pyramid logo and an online application in promoting healthy and environmentally sustainable food choices based on the MD in a worksite canteen setting during a 10-month period. Although the choice of dishes labelled with the Double Pyramid logo did not differ across the time due to a very high baseline selection, the choice of plant-based dishes, mostly those based on whole-grain cereals and legumes, and fish increased over time, while meat-based options decreased. Although the selection of healthy items increased, they were added to menus, not in replacement of other dishes, accounting for a higher level of energy and macronutrients and worse environmental footprints per meal. However, the nutritional composition of meals improved during the intervention phases. In addition, when data were adjusted for energy, no higher environmental impacts were observed, demonstrating a small improvement in the environmental sustainability of selecting menus. Therefore, the intervention was not fully effective, even though it significantly improved food choices.

Well-designed catering initiatives may offer an effective opportunity to promote healthy and sustainable food choices. However, targeted public health policy and actions are needed to support and motivate caterers to develop interventions in the desired direction. Furthermore, the study promisingly highlights the interest of consumers in receiving information in eating out scenarios about the health and environmental impact of food and demonstrates the value of using a digital application as a proper solution for users’ needs.

## Figures and Tables

**Table 1:** Number of portions (%) of food groups per tray consumed at the worksite canteens across the study and by time periods.

	Portions (*n*)/tray	Total *n* = 29,776	T0 (Jun–Sept) *n* = 7142	T1 (Sept–Dec) *n* = 10,834	T2 (Jan–Apr) *n* = 11,800	*p*-Value ^1^
Fruit	0	7719 (25.9)	2195 (30.7)	2601 (24.0)	2923 (24.8)	
	0.5	246 (0.8)	76 (1.1)	108 (1.0)	62 (0.5)	<0.001
	1	19,043 (64.0)	4225 (59.2)	7191 (66.4)	7627 (64.6)	
	1.5	87 (0.3)	54 (0.8)	27 (0.2)	6 (0.1)	
	≥2	2681 (9.0)	592 (8.3)	907 (8.4)	1182 (10.0)	
Vegetables	0	8034 (27.0)	1982(27.8)	2915 (26.9)	3137 (26.6)	<0.001
	0.5	1703 (5.7)	464 (6.5)	595 (5.5)	644 (5.5)	
	1	13,010 (43.7)	3018 (42.5)	4741 (43.8)	5251 (44.5)	
	1.5	1665 (5.6)	463 (6.5)	603 (5.6)	599 (5.1)	
	≥2	5364 (18.0)	1215 (17.0)	1980 (18.3)	2169 (18.4)	
Tubers	0	26,339 (88.5)	6357 (89.0)	9474(87.4)	10,508 (89.1)	<0.001
	1	3366 (11.3)	766 (10.7)	1348 (12.4)	1252 (10.6)	
	≥2	71 (0.2)	19 (0.3)	12 (0.1)	40 (0.3)	
Cereals	0	10,473 (35.2)	2358 (33.0)	4051 (37.4)	4064 (34.4)	<0.001
	0.5	513 (1.7)	182 (2.5)	184 (1.7)	147 (1.2)	
	1	14,200 (47.7)	3825 (53.6)	4970 (45.9)	5405 (45.8)	
	1.5	498 (1.7)	113 (1.6)	176 (1.6)	209 (1.8)	
	≥2	4092 (13.7)	664 (9.3)	1453 (13.4)	1975 (16.7)	
Whole-grain cereals	0	20,999 (70.5)	5734 (80.3)	7307 (67.4)	7958 (67.4)	<0.001
	0.5	285 (1.0)	89(1.2)	125 (1.2)	71 (0.6)	
	1	7359 (24.7)	1224 (17.1)	2942 (27.2)	3193 (27.1)	
	1.5	77 (0.3)	14 (0.2)	36 (0.3)	27 (0.2)	
	≥2	1056 (3.5)	81 (1.1)	424 (3.9)	551 (4.7)	
Pulses	0	22,088 (74.2)	5588 (78.2)	7828 (72.3)	8672 (73.5)	<0.001
	0.5	1888 (6.3)	353 (4.9)	859 (7.9)	676 (5.7)	
	1	5158 (17.3)	1064 (14.9)	1922 (17.7)	2172 (18.4)	
	1.5	248 (0.8)	45 (0.6)	99 (0.9)	104 (0.9)	
	≥2	394 (1.3)	92 (1.3)	126 (1.2)	176 (1.5)	
Dairies	0	23,748 (80.2)	5730 (80.2)	8813 (81.3)	9205 (78.0)	<0.001
	0.5	3709 (12.5)	802 (11.2)	1165 (10.8)	1742 (14.8)	
	1	2017 (6.8)	533 (7.5)	746 (6.9)	738 (6.3)	
	1.5	223 (0.7)	61 (0.9)	74 (0.7)	88 (0.7)	
	≥2	79 (0.3)	16 (0.2)	36 (0.3)	27 (0.2)	
Eggs	0	28,492 (95.7)	6783 (95.0)	10,454 (96.5)	11,255 (95.4)	<0.001
	0.5	335 (1.1)	97 (1.4)	67 (0.6)	171 (1.4)	
	1	942 (3.2)	260(3.6)	309 (2.9)	373 (3.2)	
	1.5	4 (0.0)	0 (0.0)	3 (0.0)	1 (0.0)	
	≥2	3 (0.0)	2 (0.0)	1 (0.0)	0 (0.0)	
Fish	0	22,346 (75.0)	5443 (76.2)	8059 (74.4)	8844 (74.9)	<0.001
	0.5	989 (3.3)	299 (4.2)	236 (2.2)	454 (3.8)	
	1	5900 (19.8)	1268 (17.8)	2376 (21.9)	2256 (19.1)	
	1.5	273 (0.9)	51 (0.7)	124 (1.1)	98 (0.8)	
	≥2	268 (0.9)	81 (1.1)	39 (0.4)	148 (1.3)	
Meat	0	15,756 (52.9)	3659 (51.2)	5757 (53.1)	6340 (53.7)	<0.001
	0.5	2780 (9.3)	783 (11.0)	1010 (9.3)	987 (8.4)	
	1	9905 (33.3)	2310 (32.2)	3622 (33.4)	3982 (33.7)	
	1.5	936 (3.1)	273 (3.8)	299 (2.8)	364 (3.1)	
	≥2	399 (1.3)	126 (1.8)	146 (1.3)	127 (1.1)	
Dessert	0	29,694 (99.7)	7142 (100)	10,834 (100)	11,718 (99.7)	<0.001
	1	82 (0.3)	0 (0.0)	0 (0.0)	82 (0.7)	
Beverages	0	28,696 (96.4)	6869 (96.2)	10,437 (96.3)	11,390 (96.5)	0.679
	1	1078 (3.6)	273 (3.8)	396 (3.7)	409 (3.5)	
	≥2	2 (0.0)	0 (0.0)	1 (0.0)	1 (0.0)	
Juices	0	29,767 (100)	7140 (100)	10,832 (100)	11,795 (100)	0.582
	1	9 (0.0)	2 (0.0)	2 (0.0)	5 (0.0)	

^1^ Chi-squared test.

**Table 2:** Energy and nutrient content (mean ± SD) per tray consumed at the worksite canteens across the study and by time periods.

	Total *n* = 29,776	T0 (Jun–Sept) *N* = 7142	T1 (Sept–Dec) *n* = 10,834	T2 (Jan–Apr) *n* = 11,800	*p*-Value ^1^
Energy (kcal/tray)	699 ± 208	649 ± 194 ^c^	707 ± 208 ^b^	723 ± 211 ^a^	<0.001
Proteins (g/tray)	36.3 ± 13.0	35.1 ± 12.4 ^c^	36.3 ± 12.4 ^b^	37.0 ± 13.8 ^a^	<0.001
(%Energy)	21.3 ± 7.1	22.2 ± 7.4 ^a^	21.1 ± 6.9 ^b^	20.9 ± 7.0 ^b^	<0.001
Fat (g/tray)	21.3 ± 11.7	19.6 ± 11.1 ^c^	21.6 ± 11.9 ^b^	22.1 ± 11.8 ^a^	<0.001
(%Energy)	26.7 ± 10.4	26.5 ± 10.8	26.8 ± 10.3	26.9 ± 10.3	0.052
Saturated fat (g/tray)	6.0 ± 4.9	5.6 ± 4.9 ^b^	6.1 ± 5.1 ^a^	6.1 ± 4.7 ^a^	<0.001
(%Energy)	7.3 ± 4.9	7.3 ± 5.3	7.3 ± 4.9	7.3 ± 4.6	0.762
Carbohydrates (g/tray)	88.4 ± 32.6	80.1 ± 31.1 ^c^	89.5 ± 32.3 ^b^	91.8 ± 33.0 ^a^	<0.001
(%Energy)	50.5 ± 12.7	49.7 ± 13.6 ^b^	50.7 ± 12.5 ^a^	50.8 ± 12.4 ^a^	<0.001
Sugars (g/tray)	23.3 ± 11.9	20.5 ± 11.2 ^c^	24.7 ± 12.1 ^a^	23.8 ± 11.8 ^b^	<0.001
(%Energy)	14.1 ± 7.7	13.3 ± 7.6 ^c^	14.8 ± 7.9 ^a^	13.9 ± 7.4 ^b^	<0.001
Fiber (g/tray)	11.3 ± 4.7	10.3 ± 4.4 ^c^	11.4 ± 4.7 ^b^	11.7 ± 4.9 ^a^	<0.001
(%Energy)	3.4 ± 1.7	3.4 ± 1.7 ^b^	3.4 ± 1.6 ^ab^	3.5 ± 1.7 ^a^	0.002
Salt (g/tray)	3.6 ± 1.6	3.3 ± 1.3 ^c^	3.6 ± 1.7 ^b^	3.8 ± 1.7 ^a^	<0.001

^1^ Main effect from one-way ANOVA test with Tamhane post hoc test. Different letters in the same row indicate significantly different values (*p* < 0.05).

**Table 3:** Carbon, water, and ecological footprints (mean ± SD) per tray consumed at the worksite canteens and per 1000 kcal across the study and by time period.

	Total *n* = 29,776	T0 (Jun–Sept) *n* = 7142	T1 (Sept–Dec) *n* = 10,834	T2 (Jan–Apr) *n* = 11,800	*p*-Value ^1^
Carbon Footprint (g CO_2_ eq/tray)	1112 ± 867	1061 ± 823 ^c^	1108 ± 874 ^b^	1146 ± 883 ^a^	< 0.001
Water Footprint (L/tray)	1083 ± 858	1028 ± 814 ^c^	1082 ± 879 ^b^	1117 ± 863 ^a^	< 0.001
Ecological Footprint (m^2^/tray)	8.9 ± 5.7	8.5 ± 5.4 ^b^	9.0 ± 5.7 ^a^	9.1 ± 5.8 ^a^	< 0.001
Carbon Footprint (g CO_2_ eq/1000 kcal)	1649 ± 1444	1699 ± 1474 ^a^	1626 ± 1445 ^b^	1639 ± 1423 ^b^	0.002
Water Footprint (L/1000 kcal)	1593 ± 1293	1631 ± 1324 ^a^	1575 ± 1310 ^b^	1586 ± 1256 ^b^	0.012
Ecological Footprint (m^2^/1000 kcal)	13.4 ± 9.6	13.8 ± 9.9 ^a^	13.3 ± 9.6 ^b^	13.2 ± 9.4 ^b^	< 0.001

^1^ Main effect from one-way ANOVA test with Tamhane post hoc test. Different letters in the same row indicate significantly different values (*p* < 0.05).

## Data Availability

Data are available on request due to restrictions. The data presented in this study are available on request from the corresponding author. The data are not publicly available due to privacy and company policy.

## Supplementary Materials

The following supporting information can be downloaded at: https://www.mdpi.com/article/10.3390/nu14214456/s1, Table S1: Number of portions (median (25–75th percentile)) of food groups per tray consumed at the worksite canteens across the study and by time period.
