# Supplementary material for: Nutritional Composition and Environmental Impact of Meals Selected in Workplace Canteens before and after an Intervention Promoting the Adherence to the Mediterranean Diet
Source: Nutrients. 2022 Oct 23;14(21):4456. doi: 10.3390/nu14214456 (PMC9656980; doi:10.3390/nu14214456)
Supplement: Supplementary file 1 [file nutrients-14-04456-s001.zip › Table S1_Si_Med.pdf]

Table S1. Number of portions (median (25th-75th percentile)) of food groups per tray consumed at the worksite canteens across the study and by time periods.

	Total n=30,384	T0 (June-Sept) n=7371	T1 (Sept-Dec) n=11,014	T2 (Jan-Apr) n=11,999	p-value[1] main effect
Water (portion/tray)	1.0 (1.0-1.0)	1.0 (1.0-1.0)	1.0 (1.0-1.0)	1.0 (1.0-1.0)	0.093
Beverages (portion/tray)	0.0 (0.0-0.0)	0.0 (0.0-0.0)	0.0 (0.0-0.0)	0.0 (0.0-0.0)	0.495
Juice (portion/tray)	0.0 (0.0-0.0)	0.0 (0.0-0.0)	0.0 (0.0-0.0)	0.0 (0.0-0.0)	0.579
Fruit (portion/tray)	1.0 (0.0-1.0)	1.0 (0.0-1.0)[b]	1.0 (0.5-1.0)[a]	1.0 (0.0-1.0)[a]	< 0.001
Vegetables (portion/tray)	1.0 (0.0-1.0)	1.0 (0.0-1.0)	1.0 (0.0-1.0)	1.0 (0.0-1.0)	0.180
Tubers (portion/tray)	0.0 (0.0-0.0)	0.0 (0.0-0.0)[b]	0.0 (0.0-0.0)[a]	0.0 (0.0-0.0)[b]	< 0.001
Cereals (portion/tray)	1.0 (0.0-1.0)	1.0 (0.0-1.0)[b]	1.0 (0.0-1.0)[b]	1.0 (0.0-1.0)[a]	< 0.001
Whole-grain cereals (portion/tray)	0.0 (0.0-1.0)	0.0 (0.0-0.0)[b]	0.0 (0.0-1.0)[a]	0.0 (0.0-1.0)[a]	< 0.001
Pulses (portion/tray)	0.0 (0.0-0.5)	0.0 (0.0-0.0)[b]	0.0 (0.0-0.5)[a]	0.0 (0.0-0.5)[a]	< 0.001
Dairy products (portion/tray)	0.0 (0.0-0.0)	0.0 (0.0-0.0)[b]	0.0 (0.0-0.0)[b]	0.0 (0.0-0.0)[a]	< 0.001
Egg (portion/tray)	0.0 (0.0-0.0)	0.0 (0.0-0.0)[a]	0.0 (0.0-0.0)[b]	0.0 (0.0-0.0)[a]	< 0.001
Fish (portion/tray)	0.0 (0.0-0.0)	0.0 (0.0-0.0)[b]	0.0 (0.0-0.5)[a]	0.0 (0.0-0.0)[ab]	0.004
White meat (portion/tray)	0.0 (0.0-0.0)	0.0 (0.0-0.0)[a]	0.0 (0.0-0.0)[b]	0.0 (0.0-0.0)[c]	< 0.001
Red meat (portion/tray)	0.0 (0.0-0.0)	0.0 (0.0-0.0)[b]	0.0 (0.0-0.0)[a]	0.0 (0.0-0.0)[a]	< 0.001
Cured meat (portion/tray)	0.0 (0.0-0.0)	0.0 (0.0-0.0)[a]	0.0 (0.0-0.0)[b]	0.0 (0.0-0.0)[b]	< 0.001
Desert (portion/tray)	0.0 (0.0-0.0)	0.0 (0.0-0.0)[b]	0.0 (0.0-0.0)[b]	0.0 (0.0-0.0)[a]	< 0.001

[1] Kruskal-Wallis test with pairwise comparisons. Different letters in the same row indicate significantly different values ($p < 0.05$).
